# Otological Planning Software—OTOPLAN: A Narrative Literature Review

**DOI:** 10.3390/audiolres13050070

**Published:** 2023-10-18

**Authors:** Annalisa Gatto, Margherita Tofanelli, Ludovica Costariol, Serena Rizzo, Daniele Borsetto, Nicoletta Gardenal, Francesco Uderzo, Paolo Boscolo-Rizzo, Giancarlo Tirelli

**Affiliations:** 1 Department of Medical, Surgical and Health Sciences, Section of Otolaryngology, University of Trieste, 34149 Trieste, Italy; annalisa.gatto@asugi.sanita.fvg.it (A.G.); nicoletta.gardenal@asugi.sanita.fvg.it (N.G.); tirellig@units.it (G.T.); 2 Department of ENT, Addenbrookes Hospital, Cambridge University Hospitals NHS Foundation Trust, Cambridge CB2 0QQ, UK

**Keywords:** cochlear implant, OTOPLAN, otological planning software, otosurgery

## Abstract

The cochlear implant (CI) is a widely accepted option in patients with severe to profound hearing loss receiving limited benefit from traditional hearing aids. CI surgery uses a default setting for frequency allocation aiming to reproduce tonotopicity, thus mimicking the normal cochlea. One emerging instrument that may substantially help the surgeon before, during, and after the surgery is a surgical planning software product developed in collaboration by CASCINATION AG (Bern, Switzerland) and MED-EL (Innsbruck Austria). The aim of this narrative review is to present an overview of the main features of this otological planning software, called OTOPLAN^®^. The literature was searched on the PubMed and Web of Science databases. The search terms used were “OTOPLAN”, “cochlear planning software” “three-dimensional imaging”, “3D segmentation”, and “cochlear implant” combined into different queries. This strategy yielded 52 publications, and a total of 31 studies were included. The review of the literature revealed that OTOPLAN is a useful tool for otologists and audiologists as it improves preoperative surgical planning both in adults and in children, guides the intraoperative procedure and allows postoperative evaluation of the CI.

## 1. Introduction

Conventional acoustic hearing aids become less effective for individuals affected by severe to profound sensorineural hearing loss. For a subset of this population, the emergence of cochlear implants (CIs) has offered an alternative therapeutic option [1].

A CI translates acoustic signals into electrical impulses that are then conveyed to the organ of Corti in the cochlea. The regular cochlea has a tonotopic map that conducts characteristic frequencies (CFs) to precise locations along the basilar membrane (BM).

CFs correspond to the frequency at which the cochlear BM expresses the highest sensitivity with the lowest sensation sound level [2].

The arrangement of frequencies across space is known as cochlear tonotopy. Nerve fibers extend from the BM to the spiral ganglion, which is situated nearer to the central axis of the cochlear spiral and houses the cell bodies of neurons [3].

To achieve the optimal functional outcome for CI patients, it is expected that tonotopic stimulation should be as precise as possible, meaning that the CI electrodes stimulate in accordance with their postoperative tonotopic positions [4].

The variation in post-surgical speech perception can be affected by the patient’s own physiologic features such as severity of deafness, age at CI surgery, etiology of hearing loss, degree of residual hearing, and several other preimplantation cognitive factors [5,6]. While these variables are non-modifiable, there are other controlling factors able to impact outcomes that could affect the allocation of the CI electrodes inside the cochlea. Among them, the length and type of the electrode and the depth of insertion/angular insertion depth (AID) [7] could be regulated in the surgical planning process and integrated in tool mapping procedures. The maximum benefit for patients is achieved when the electrode array is positioned optimally and completely within the scala tympani (ST) [8,9] (see the Video S1 in the supplemental material section). Scala vestibuli (SV) insertion is possible in special cases of ST ossification [10] without any degree of scalar transposition, thus creating an effective electrode–neural interface [11]. In particular, previous studies have proposed that gradual stable insertion could lower pressure fluctuations within the cochlea, decrease inset strengths, increase the chance of an in-axis insertion into the ST, and ameliorate hearing results [12].

Currently, standard CI surgery uses a default setting for frequency allocation that aims to replicate tonotopicity, thus mimicking the normal cochlea. In the CI, electrode arrays are surgically placed within the cochlea to directly activate nerve fibers through electrical signals, circumventing the impaired sensory cells and mechanoelectrical transduction process [3]. This procedure consists of displaying the more basal electrodes of the arrays to activate the higher-frequency section and the more apical electrodes to stimulate the lower-frequency region [13].

The frequency-to-place matching of the CI electrode connection is usually based on the Greenwood function [14]. This technique assigns a CF to the acoustic nerve fibers based on the location of its peripheral tip along the BM [15]. In some cases, the pre-set frequency-to-electrode distribution is assigned following the Greenwood function, regardless of individual distinctions [16].

Electrode arrays come in several different designs that influence the allocation of the CI electrodes within the cochlea [17]; when using the default frequency programming, the variety in electrode form typically affects the particular region of the cochlea being stimulated. The correlation of the electrode array to the microanatomy of the cochlea can impact on auditory performance in some CI recipients. Indeed, a recent review provided an extensive summary of the available electrode materials and shapes, along with their impact on stimulation; the authors also addressed the different mechanical properties, investigating the stiffness and flexibility of the electrode arrays [18,19]. One important factor that may affect hearing quality is the ability to stimulate as many cochlear cells as possible; nowadays, the available designs of the electrode arrays allow a noisy auditory perception because of the electric fields’ interaction. To improve the channel interactions, the electrode array designs should be rigid and flexible enough to be inserted without tissue damage and with minimal trauma [19]. Emerging research in the literature has shown that the cochlear duct length (CDL) could be considered in preoperative settings to better choose the appropriately sized electrode and to provide customized frequency maps, but how exactly this can influence speech outcomes is unclear [17,20,21].

When pre-set frequency programming is applied, differences in the type of implant, CDL, and degree of insertion may cause discrepancies between the CF of the cochlea and the frequency that the electrode presents to that part of the cochlea, leading to an allocation mismatch. [16] To solve the allocation mismatch, the gap between the default frequency setting and an anatomy-based frequency allocation in CI electrode anatomy-based fitting was investigated [22,23].

At this stage, it became necessary to implement a system that could facilitate a personalized examination of the patient’s anatomy before the scheduled surgery, rather than relying on a standard approach to guide the surgical preparations for CI.

One emerging instrument that may help the surgeon before, during, and after the surgery is an otological planning software product created in cooperation by CASCINATION AG (Bern, Switzerland) and MED-EL (Innsbruck Austria) [6]. This system, called OTOPLAN^®^, allows three-dimensional handling that can be used preoperatively by the otologist to study the cochlear anatomy and visualize the electrode insertion depth prior to surgery, and to choose the best electrode array for that patient; postoperatively, it can be applied to check frequency allocation, and the proper CI position.

The aim of this narrative review is to describe the main features of the OTOPLAN^®^ otological planning software and its different uses and to discuss the advantages and disadvantages of its use in CI surgery.

## 2. Materials and Methods

The literature was searched on the PubMed and Web of Science databases in May 2023. No time limitations were applied. The search terms were “OTOPLAN”, “cochlear planning software” “three-dimensional imaging”, “3D segmentation”, and “cochlear implant” combined into the following query: ‘cochlear planning software’ * AND [(‘cochlear implant*’ OR ‘cochlear implant surgery*’ OR ‘otosurgery’) AND (‘3D segmentation’ OR ‘three-dimensional imaging’ OR ‘OTOPLAN’)]. Papers published until the date of the review that contained this query in the title or the abstract were selected. We limited our search to publications indexed as articles, proceedings or reviews available in the English language.

## 3. Results

This strategy yielded 52 publications. We excluded 4 articles that were focused not on CI but on other types of middle ear implants or bone-conduction implants; 5 articles that did not use the OTOPLAN^®^ system but used alternative software; and 8 articles that focused on robotic surgery for CI with or without OTOPLAN^®^. Finally, a total of 31 studies were included, among which 15 focused on adults only and 13 focused on children or mixed populations of adults and children, whereas 3 did not provide information on the age of the patients. Table S1 summarizes the main features of the studies included in the review.

## 4. Discussion

This review included several studies addressing the use of OTOPLAN^®^ as a tool for preoperative surgical planning and postoperative electrode placement analysis and anatomy-based fitting in CI surgery.

### 4.1. What Is OTOPLAN and How Does It Work?

OTOPLAN, a standalone software tool, enables the generation of 3D reconstructions from digital imaging and communications in medicine (DICOM) images, offering a multifaceted approach to cochlear parameter calculation and visualization. This software not only facilitates the visualization of postoperative electrode positions but also ensures individualized matching and provides a comprehensive patient report, alongside a detailed postoperative analysis [24].

**Figure 1:**
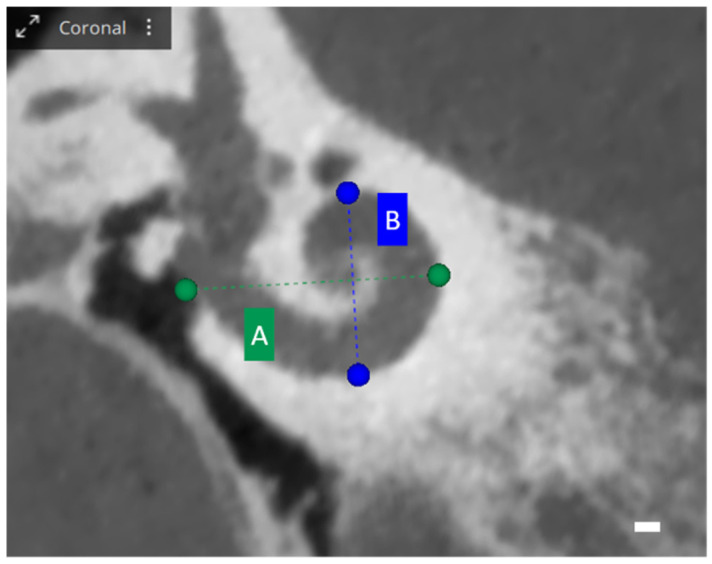
OTOPLAN cochlear parameters, screenshot of a right cochlea. Coronal view: the blue and green lines show the diameter A and the width B of the basal turn. Scale bar: 1 mm.

**Figure 2:**
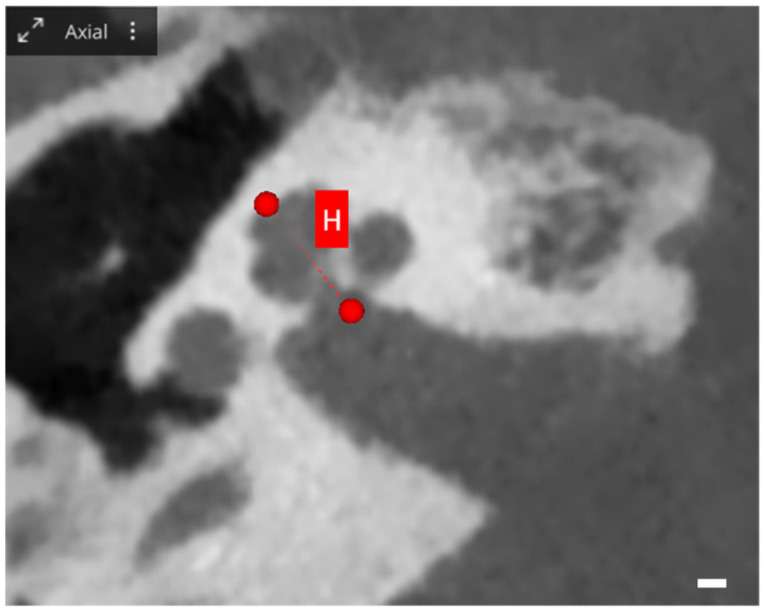
OTOPLAN cochlear parameters, screenshot of a right cochlea. Axial view: the red line shows the height H, which represents the height of the cochlea, the distance between the center of the basal turn and the superior apex point. Scale bar: 1 mm.

When exploring cochlear metrics with OTOPLAN, several values are assessed, each providing unique insights into cochlear dimensions. The A-value represents the diameter, defined as the maximum distance from the round window to the lateral wall of the basal turn, passing through the modiolus. Meanwhile, the B-value illustrates the width of the cochlea, measured perpendicularly to the A-value line at the modiolus. Additionally, the H-value indicates the height of the cochlea, quantifying the space between the center of the basal turn and the apex point. These metrics are visually represented in Figure 1 and Figure 2 [25].

OTOPLAN adeptly localizes the modiolus, round window, and cochlear boundaries to calculate the CDL at the organ of Corti level, utilizing the elliptic–circular approximation (ECA) method [25]. Historically, CDL estimation has been approached through various methods, including direct and indirect strategies and 3D reconstructions, each employing a calculation of spiral coefficients with differing accuracy levels [26,27].

Given that cochlear dimensions can fluctuate significantly, ranging from 19.71 to 45.6 mm and potentially varying by 30% to 40% [23,28], it is imperative for surgical teams to preoperatively consider the interindividual variability in normal cochlear morphology and other parameters. This ensures the selection of the most suitable electrode type, capable of adapting to potential anatomical variations both in normal anatomical conditions and in cases of cochlear malformations [29].

The capability of OTOPLAN in determining both the angular insertion depth (AID) of the CI [30] and the CDL has been validated and deemed comparable to that of other methods [31]. The software not only provides CDL dimensions but also, utilizing the Greenwood mathematical function [14], generates an individual frequency map. This map pinpoints the exact intracochlear site where neural fibers process various sound signal frequencies.

Accurate cochlear dimension estimates assist surgeons in selecting the most apt electrode array, ensuring an optimal length for each specific patient. This precision proves particularly insightful in instances of inner ear malformations and during the postoperative activation phase, where the frequency map can be utilized to enhance frequency reallocation.

Designed to be compatible with both CT and MRI scans, OTOPLAN has demonstrated a minimal impact of CT slice thickness on CDL measurement [18]. Furthermore, its performance in estimating CDL has been shown to be comparably effective when using MRI or the gold-standard CT scans [32].

### 4.2. Different Uses of OTOPLAN

#### 4.2.1. Anatomical Study

The dimensions and form of the cochlea influence CI electrode placement and cochlear coverage, affecting final pitch discrimination [8,24]. The CDL impacts CI outcomes, highlighting a need for more research to improve patient-customized surgical planning and postoperative mapping techniques. However, a reliable method for assessing detailed cochlear dimensions is yet to be identified [21,33,34].

Utilizing CT imaging and 3D reconstruction, OTOPLAN has been applied in various research contexts to explore ear anatomy [35]. The software has demonstrated safety for in-depth studies of children with different cochlear structures [36,37] and has been validated through comparisons with the clinical DICOM viewer [23].

The research indicates no difference in the A-value measured via DICOM and OTOPLAN, affirming its applicability for pediatric patients. Furthermore, a notable correlation exists between cochlear parameters (A-value, B-value, and H-value) and the CDL, with a combination of the A-value and B-value providing a precise CDL estimation. Additionally, men generally present higher cochlear parameters than women, with CDL measurements being comparable to those obtained through the use of alternative techniques [23,31,38].

While OTOPLAN’s reliability in estimating cochlear parameters has been acknowledged, concerns about its accuracy in malformed ears have been raised [18]. The software has been used to develop methods for evaluating the cochlear basal turn and identifying inner ear malformations [36].

OTOPLAN’s measurements of cochlear parameters in children were used in 3D segmentation software to measure the vestibular aqueduct (VAD) volume. A strong correlation was found between OTOPLAN measurements and VAD volumes, enabling the development of a system to calculate both CT inner ear volume and VAD volumes, particularly in subjects with an enlarged vestibular aqueduct requiring CI surgery. Gender-based differences in inner ear anatomy were also confirmed, and gender and VAD width at the midpoint were identified as significant predictors of the risk of gushing [39].

Measuring the height of the scala tympani is challenging due to the limited resolution of clinical CT scans. OTOPLAN facilitates the generation of personalized, oblique cochlear views using available CT images [40].

Research using OTOPLAN to explore the relationship between mastoid thickness and growth from 6 months to 20 years revealed a logarithmic growth in mastoid thickness that plateaus at 20 years. This information can assist otosurgeons in determining the required drilling depth in the mastoid and potentially inform improvements in CI design to minimize excess electrode lead length [41].

A retrospective evaluation compared cochlear parameters measured manually by two expert otosurgeons with those automatically obtained using a development version of the OTOPLAN^®^ software. The evaluation noted excellent inter-rater reliability, a high agreement of outcomes, and a reduction in execution time using the automatic method [42].

#### 4.2.2. Surgical Planning

The pivotal role of OTOPLAN in CI surgery is illuminated through its utility in assisting surgeons in devising a strategic surgical approach, foreseeing potential intraoperative complications, and choosing an electrode array that harmonizes with the specific cochlear anatomy. A variety of research has explored these aspects, investigating diverse methodologies for segmenting temporal bone surgical anatomy for patient-specific virtual reality simulation. Automated segmentation algorithms have emerged as a flexible and viable approach, particularly useful in managing cases with abnormal anatomy [43].

A notable concern in CI surgery is intraoperative complications, which can lead to endoluminal fibrosis and round window ossification. Ensuring complete array insertion is crucial, not only to secure an optimal tonotopic match but also to stimulate the apex of the cochlea, which is vital for speech understanding and music perception, and where mismatch is notably pronounced [44].

Moreover, OTOPLAN has proven effective in instances of severely advanced otosclerosis, including those with cochlear ossification and anatomical abnormalities, as well as in cases with a normally shaped cochlea, where conventional DICOM viewers were insufficient in providing necessary information. Particularly, OTOPLAN has demonstrated its worth in challenging cases of severely advanced otosclerosis, with comparative studies revealing that patients who underwent CI surgery with OTOPLAN evaluation exhibited slightly better outcomes and encountered no instances of incomplete array insertion, as opposed to a historical group where surgical planning was conducted using CT images [45,46,47].

Effective surgical planning, which enables the surgeon to sidestep critical issues during CI surgery, should be accomplished using the least invasive approach. Utilizing OTOPLAN-reconstructed imaging provides insightful analysis of the optimal surgical trajectory line through the retro-facial recess towards the round window and also enables the measurement and classification of the size of the facial recess, comparing the retro-facial approach to the standard facial recess approach [48].

Accuracy in estimating the CDL is augmented by three-dimensional reconstructions, which scrutinize the entire 3D structure of the cochlea and are not subject to viewing angle effects, thereby representing the most precise technique for CDL estimation [49].

Choosing the correct electrode array length is another crucial aspect in CI surgery, given the array of electrodes available, which vary in length and structure. OTOPLAN has been utilized to measure the CDL and estimate the frequency allocation map, assisting in selecting the most fitting electrode array [50]. In pediatric CI surgery, it has been observed that the physiological function of the peripheral auditory system is influenced by the anatomical structure of the cochlea, with a larger cochlear size being associated with better auditory conduction function [51].

OTOPLAN has also been validated in determining the postoperative AID, CDL, and cochlear place frequency with a lateral-wall array [30,52]. AID and CDL, using the Greenwood function, provide valuable information about electrode location in relation to the tonotopic organization of the cochlea, which is useful to inform image-guided mapping strategies for CI recipients postoperatively. A deeper electrode insertion has been associated with an improvement in the audiologic outcome [53,54]. The determination of the AID provides crucial prognostic information due to the relationship between insertion depth and speech perception in CI recipients, allowing clinicians to measure the AID with lateral-wall arrays quickly and reliably, reducing the mismatch between the electrically presented frequency information and the cochlear place frequency [54].

Furthermore, it has been suggested that the customized placement of slim modiolar electrodes, based on CDL measurements using OTOPLAN, can result in improved modiolar proximity [55]. A shorter CDL has been linked to a less tightly coiled arrangement of slim modiolar electrodes, which may be influenced by the insertion technique. Therefore, the varying levels of modiolar proximity with slim modiolar electrodes can potentially be mitigated in cases with shorter CDL measurements. Future studies have been suggested to estimate other variations in cochlear morphology that could predict resistance and failure to achieve complete insertion with long arrays [56].

#### 4.2.3. Postoperative Frequency Reallocation

Ensuring a complication-free cochlear implant procedure involves a meticulous preoperative assessment, which aims to facilitate atraumatic insertion and address individual anatomical variations. Nonetheless, achieving optimal audiological outcomes can be intricate due to patient-specific factors, unpredictable comorbidities, and potential surgical complications [57].

The complexity of cochlear differences is well-established. Even when the cochlear morphology seems normal on CT scans, surgical challenges, such as partial insertion due to a smaller cochlea or injury to the lamina spiralis, can arise because traditional CT scans may not be sufficiently sensitive to identify certain issues [31].

In the postoperative phase, the use of OTOPLAN allows surgeons to visualize the electrode insertion status from postoperative CT scans, enabling the application of an anatomy-based fitting map. This map, utilizing patient-specific data, aims to provide a fitting that closely aligns with natural tonotopic perception, considering critical parameters like the calculated tonotopic frequency of each electrode contact, determined by its angular location [58].

In a comparative analysis of two insertion techniques using OTOPLAN, no differences were found in electrode array position or position in the scala tympani, regardless of whether insertion was through the round window or through anteroinferior cochleostomy in the transcanal Veria technique [59]. Another application of OTOPLAN in postoperative follow-up involved measuring the cochlear length, lateral wall insertion angle, and insertion depth, and then estimating the tonotopic mismatch. It was observed that smaller cochlea sizes correlated with a higher insertion angle and a lesser tonotopic mismatch, suggesting that preoperative measurement of individual cochlear parameters could effectively guide electrode array choice to minimize tonotopic mismatch [44].

Investigations into the impact of existing frequency-to-place mismatch on speech perception in noise have indicated that a smaller mismatch correlates with improved speech perception in noise after 6 months. However, it has been acknowledged that additional research is needed to explore tonotopic fitting strategies based on postoperative CT images, revealing precise electrode contact positions [60,61].

Analyzing a postoperative CT using otological planning software allows for creating an anatomy-based frequency map, aiming to reallocate electrode array frequency and minimize mismatch. A comparison between anatomy-based and default frequency allocations revealed significant differences, seemingly related to cochlear coverage [16].

A recent study investigated the impact of frequency reallocation based on an anatomical frequency map on speech perception and observed an improvement in speech perception, particularly in the parameters of Speech Recognition Threshold and Speech Awareness Threshold, following the reallocation [58].

#### 4.2.4. Advantages and Disadvantages

OTOPLAN software serves as a preoperative tool for surgeons, assisting in the selection of a more specific electrode array to optimize speech perception and facilitate postoperative anatomy-based fitting. A consensus regarding its utility in automatic cochlear measurements (CDL and frequency mapping) of A-, B-, and H-values has been noted, enabling a more personalized approach to cochlear implant (CI) surgery [23,38].

Leveraging automated tools and 3D visualization, the system enables quicker and more precise measurements compared to manual methods using the conventional DICOM viewer. This enhancement in measuring cochlear parameters and automatically calculating the CDL could potentially streamline clinical workflows and provide otosurgeons with greater autonomy. The precision of software-based cochlear reconstructions, even for malformed inner ears, has been substantiated, with no device-related complications reported in the existing literature [36].

However, a few aspects of OTOPLAN deserve comment. Firstly, it necessitates that surgeons, audiologists, and radiologists become acquainted with the procedure, implicating a learning curve and potential variability in measurements due to differing training experiences [18]. The latest version of OTOPLAN has addressed this by introducing an automatic cochlear measurement tool, which offers reliable results with diminished execution time, even for users with limited radiological experience [42]. Secondly, the postoperative analysis of electrode insertion status relies on a postoperative CT scan, which is not a standard practice without OTOPLAN. Consequently, its application is restricted in pediatric populations due to concerns about radiation exposure [32].

The latest OTOPLAN version also introduces the capability to merge CT and MRI images, enhancing preoperative evaluation. Furthermore, integrating OTOPLAN with intraoperative electrophysiologic testing could potentially mitigate misplacement and establish correlations with postoperative acoustic hearing, presenting an additional benefit.

## 5. Conclusions

A review of the existing literature underscores the utility of OTOPLAN for otologists and audiologists, enhancing preoperative surgical planning for both adults and children by providing detailed anatomical information, guiding intraoperative procedures, and facilitating postoperative evaluation of cochlear implantation. Notably, the system enables automatic CDL calculation, frequency mapping using A and B diameter measurements, and assistance in electrode selection. In postoperative stages, OTOPLAN provides a visualization of the electrode insertion status through postoperative CT scans, allowing audiologists to utilize an anatomy-based fitting map. This map uses patient-specific data to closely approximate natural tonotopic perception. However, additional research is warranted to refine the accuracy of CDL estimation, which is crucial for a thorough evaluation of pitch match and cochlear coverage in residual hearing.

## Data Availability

Data sharing not applicable. No new data were created or analyzed in this study. Data sharing is not applicable to this article.

## Supplementary Materials

The following supporting information can be downloaded at: https://www.mdpi.com/article/10.3390/audiolres13050070/s1.
