# Supplementary material for: Otological Planning Software—OTOPLAN: A Narrative Literature Review
Source: Audiol Res. 2023 Oct 18;13(5):791–801. doi: 10.3390/audiolres13050070 (PMC10603892; doi:10.3390/audiolres13050070)
Supplement: Supplementary file 1 [file audiolres-13-00070-s001.zip › Table S1.pdf]

Author	Title	Publication year	State	Study design	N° of patients	Age	Disease	Group of patients	Cochlear Implant	Objective	OTOPLAN analysis	Outcome analyzed	Results
Lovato *et al.*[40]	OTOPLAN in Cochlear Implantation for Far-advanced Otosclerosis	2020	Europe, Italy	Retrospective	13	mean 59.6	Far-advanced otosclerosis	OTOPLAN group: 5 pz Historical group (only CT pre-op): 8 pz	Unilateral	To preliminary evaluate the potential role of OTOPLAN for electrode length choice, or to predict surgical difficulties in FAO	Cochlear duct length: mean 32,4 mm Ossification/fibrosis of the cochlear duct: 2/5 Round window (RW) ossification: 2/5	Surgical difficulties: RW ossification; incomplete array insertion Adverse effects: facial palsy/stimulation; vertigo Speech recognition threshold (SRT) Words recognition score (WRS)	The audiological outcome of the OTOPLAN group was slightly superior compared with historical group. CT was not able to predict surgical difficulties; OTOPLAN was able to identify preoperatively RW niche ossification. No incomplete array insertion in OTOPLAN group; 25% in CT group
Ricci *et al.* [41]	OTOPLAN, Cochlear Implant, and Far-Advanced Otosclerosis: Could the Use of Software	2022	Europe, Italy	Case report	1	73	Far-advanced otosclerosis	/	Unilateral	Case of FAO with destruction of cochlear turns: OTOPLAN used to plan the surgery, identify the	Diameter, Height, Width, CDL of the cochlea Ossification/fibrosis of the CD Ossification of the RW	Verification of successful insertion of electrode: intraoperative radioscopy and post-operative CT	Successfully implanted a severe case of FAOwith several perforation of the cochlea.

	Improve the Surgical Final Indication?									best electrode annd check the correct position of the CI in the cochlear duct	Osteolytic areas	Intraoperative telemetry	
Lovato *et al.* [39]	Utility of OTOPLAN Reconstructed Images for Surgical Planning of Cochlear Implantation in a Case of Post-meningitis Ossification	2019	Europe, Italy	Case report	1	46	Post-meningiditis HL e cochlear ossification	/	Bilateral	To demonstrate the use of OTOPLAN for pre-operative surgical planning in a case where as according to CT surgery was contraindicated	Ossification of the RW<br>Ossification of the CD<br>CDL	Completed array insertion<br>Intraoperative telemetry	Successfully implanted. OTOPLAN may be useful in difficult CI case as cochlear ossification and anatomic abnormalities
Hajr *et al.*[42]	Cochlear Implantation: The use of OTOPLAN Reconstructed Images in Trajectory Identification	2023	Saudi Arabia	Retrospective	25	1-50	/	/	Unilateral	To define the best electrode trajectory line in cochlear implant surgery by using OTOPLAN. To investigate the feasibility of the retro-facial approach as a direct access to the RW and	Cochlear view and other ear structures.<br>Facial recess size	Ideal trajectory line<br>Size of the retrofacial approach	The retro-facial approach represented the best trajectory line. OTOPLAN-reconstructed imaging provided a useful analysis of the retro-facial approach and helped in planning the surgical trajectory

										to classify the size of the retro-facial approach in relation to the size of the facial recess.			line toward the RW. Additionally can help the surgeon to compare the retro-facial approach to the standard facial recess.
Di Maro *et al.*[52]	Frequency reallocation based on cochlear place frequencies in cochlear implants: a pilot study	2022	Europ e, Italy	Retrospect ive/ Pilot study	10	>14 (range 14.3–78.7 years)	Postlingual deafness	Pre e post frequency reallocatio n	Unilatera l	to demonstrat e that an anatomicall y nased frequency reallocation can provide immediate benefit to the patient	Array insertion depht<br>Cochlear place of stimulation	Subjective sensations<br>PTA, SAT and SRT thresholds<br>Correlation between depth of insertion and audiological thresholds	The mean values of SRT and SAT were significantly lower. No significant differences in PTA. OTOPLAN may be used in anatomic mapping for subsequent frequency reallocation
Khurayzi *et al.*[19]	Direct measureme nt of cochlear parameters for automatic calculation of the cochlear duct length	2020	Saudi Arabia	Retrospect ive	88 cochle as	<7	Prelingual deafness	CT measurem nets<br>OTOPLAN measurem ents	Uni/Bilat eral	Validation of OTOPLAN and CDL estimation	Cochlear diameter (A-value)<br>Width of the cochlear base (B-value)<br>Height of the cochlea (H-value)<br>CDL	A-value, B-value, H-value and CDL	No difference in A-value between CT and OTOPLAN<br>Both A- and B- values showed a high positive correlation to the CDL,

													stornger between B-value anCDL than A-value and CDL.
Dahanasing *et al.*[44]	The rationale for FLEX (cochlear implant) electrode with varying array lengths	2021	Europe, Austria	Narrative review	/	/	/	/	/	Rationale of FLEX electronde array and the uso of otological pre.planning software tool like OTOPLAN	/	/	OTOPLAN offers the possibility in measuring the the cochlear size, choosing the best fitting electrode array .
Spiegel *et al.*[23]	Variation of the cochlear anatomy and cochlea duct length: analysis with a new tablet-based software	2022	Europe, Germany	Retrospective	108	6,5-90,3	/	/	Uni/Bilateral	To evaluate the range of CDL, find differences in different patient groups and to assess the angular insertion depht)AID) for cochlear coverage.	Cochlear diameter (A-value)<br>Width of the cochlear base (B-value)<br>Height of the cochlea (H-value)<br>CDL<br>AID<br>Cochlear place frequency	Cochlear diameter (A-value)<br>Width of the cochlear base (B-value)<br>Height of the cochlea (H-value)<br>CDL<br>AID<br>Cochlear place frequency	Significant difference of mean CDL with regards to sex, but not to age, side or patients having received different types of CI-electrodes. Significant differences in AID and cochlear coverage.

Alahmadi *et al.*[33]	Cochlear Implantation: The Volumetric Measurement of Vestibular Aqueduct and Gusher Prediction	2023	Saudi Arabia	Retrospective chart review	21	mean 13,81 (+-5,10)	Mondini dysplasia and enlarged vestibulare aqueduct	/	Uni/Bilateral	To validate the role of 3D segmentation in measuring the volume of the vestibula aqueduct, and the inner ear and to study the correlation between VAD volume and VAD linear measurements at the midpoint and opercolum	Cochlear diameter (A-value)<br>Width of the cochlear base (B-value)<br>Height of the cochlea (H-value)<br>CDL<br>VAD widths	Cochlear diameter (A-value)<br>Width of the cochlear base (B-value)<br>Height of the cochlea (H-value)<br>CDL<br>VAD widths	Age, H-value, VAD at the midpoint and VAD at the operculum were significant predictors for CT VAD volume. Sex, age, A-.value, and VAD at the operculum ca be used as significant predictors for Ctinner ear volume using data from both ears. Patients gusher outcomes were significantly differentiated by sex and VAD lenght at the midpoint.
George-Jones *et al.*[24]	Comparing Cochlear Duct Lengths Between CT and MR Images	2020	United States of America	Retrospective	27 cochleas	adult (unspecified age)	Postlingual deafness	CDL using CT and CDL using MR	Uni/Bilateral	To examine the intra- and interoserver variability in measunring the CDL	CDL	CDL	No difference between MRI and CT: MRI images can be used in OTOPLAN

	Using an Otological Surgical Planning Software									from MRI images vs CT images using the OTOPLAN			as CT images.
Canfarotta *et al.*[22]	Validating a new tablet-based tool in the determination of cochlear implant angular insertion depth	2019	United States of America	Retrospective	36 cochleas (20 pz)	adult (unspecified age)	Postlingual deafness	/	Uni/Bilateral	To evaluate the intra- and inter-retar reliability of this tool in determining AID and CDL. To assess the resultant variability in estimates of the cochlear place frequency for the most apical electrode.	AID<br>CDL<br>Estimate cochlear place frequency for each electrode	AID<br>CDL<br>Estimate cochlear place frequency for each electrode	Excellent inter- and inter rater reliability of both AID and CDL: OTOPLAN can be used to reliably determine electrode location to inform image-guided mapping strategies for CI recipients.
Bahavana *et al.*[53]	OTOPLAN-Based Study of Intracochlear Electrode Position Through Cochleostomy and Round Window in Transcanal Veria Technique	2022	India	Retrospective	26	2-15	Prelingual deafness; severe bilateral HL	/	Uni/Bilateral	To study the postoperative visualisation of the electrode array insertion angle through transcanal Veria approach in both round window and cochleostomy tech-niques	Cochlear diameter (A-value)<br>Height of the cochlea (H-value)<br>Width of the cochlear base (B-value)<br>CDL	Cochlear diameter (A-value)<br>Height of the cochlea (H-value)<br>Width of the cochlear base (B-value)<br>CDL	No significant difference in average angle of insertion depth between subjects with cochleostomy and round window insertion. No difference between round

													window insertion or cochleostomy insertion when it comes to electrode array position and placement in the scala tympani.
Cooperman *et al*.[14]	Assessment of Inter- and Intra-Rater Reliability of Tablet-Based Software to Measure Cochlear Duct Length	2021	United States of America	Retrospective	166 cochlea (83 pz)	mean 65.63	/	/	Uni/Bilateral	To examine whether increased CT slice thickness was associated with increased variability of CDL measurements.<br>To assessed the strong inter- and intra-rater reliability.	Cochlear diameter (A-value)<br>Width of the cochlear base (B-value)<br>CDL	Cochlear diameter (A-value)<br>Width of the cochlear base (B-value)<br>CDL	No significant relationship between slice thickness and CDL measurement. There is inter- and intra-rater reliability for cochlear diameter, width, and duct length measured with OTOPLAN. The software may have clinical utility for selecting appropriate electrode array lengths.

Dutrieux *et al.*[38]	Correlation Between Cochlear Length, Insertion Angle, and Tonotopic Mismatch for MED-EL FLEX28 Electrode Arrays	2022	Europe, France	Retrospective	106 cochleae (99 pz)	mean 63	Severe to profound HL	Size of CDL: Small <33.3mm Medium 33.3-36.2mm Large > 36.2mm	Uni/Bilateral	To investigate the relationship between cochlear length, insertion angle, and tonotopic mismatch and to compare the tonotopic mismatches with respect to the spiral ganglion and the organ of Corti.	Cochlear diameter (A-value)<br>CDL<br>AID	Cochlear diameter (A-value)<br>CDL<br>AID	Small cochlea size corresponded to higher insertion angle. Tonotopic mismatch could be minimized preoperatively by choosing electrode arrays according to the individual cochlear morphology and postoperatively by appropriate frequency fitting.
Chen *et al.*[32]	Cochlear Duct Length Calculation: Comparison Between Using Otoplan and Curved Multiplanar Reconstruction in Non malformed Cochlea	2021	China	Retrospective	68 cochleae (34 pz)	0,6-63,3	/	/	/	To describe a new method to measure the cochlear parameters using Otoplan software, and to compare it with the traditional method using curved multiplanar	Cochlear diameter (A-value)<br>Height of the cochlea (H-value)<br>Width of the cochlear base (B-value)<br>CDL<br>Depth of insertion<br>Frequency corresponding electrode	Cochlear diameter (A-value)<br>Height of the cochlea (H-value)<br>Width of the cochlear base (B-value)<br>CDL<br>Depth of insertion<br>Frequency corresponding electrode	Length, width, height, and CDL measured by Otoplan, showed no significant differences compared with measurements made by cMPR. CDL presented statistically significant

										reconstruction (cMPR).			differences between male and female patients. Measurements using Otoplan presented better repeatability and are much rapid.
Lee *et al.*[49]	Modiolar Proximity of Slim Modiolar Electrodes and Cochlear Duct Length: Correlation for Potential Basis of Customized Cochlear Implantation With Perimodiolar Electrode	2021	South Corea	Retrospective	51 cochlea (38 pz)	7-91	Congenital deafness Postlingual deafness	Congenital vs deafness Modiolar proximity: less vs tight	Unilateral	To evaluate individual CDL to determine if there is any significant correlation of CDL with degree of modiolar proximity	Cochlear diameter (A-value) Height of the cochlea (H-value) Width of the cochlear base (B-value) CDL	Relationships between cochlear parameters and spiral diameters	A preponderance of less modiolar proximity of the electrode exclusively among congenital deafness cases. Shorter CDL is associated with a less tight spiral configuration of slim modiolar electrodes postoperatively.
Aljazeeri *et al.*[12]	Anatomy-based frequency allocation in cochlear implantation: the	2022	Saudi Arabia	Retrospective	169 cochlea (102 pz)	mean 7 (+-10) range:10 month-74years	/	default frequency setting avs anatomy based frequency	Uni/Bilateral	This study aimed to compare the predicted anatomy-based	Cochlear diameter (A-value) Height of the cochlea (H-value) Width of the	Frequency-to-place mismatch	The anatomy-based frequency allocation of each electrode is

	importance of cochlear coverage							reallocation		frequency allocation of cochlear implant electrodes with the default standard frequencies.	cochlear base (B-value)<br>CDL		significantly different from the default frequency setting. This frequency-to-place mismatch was affected mainly by the cochlear coverage.
Paouris *et al.*[36]	Validation of Automatic Cochlear Measurement using OTOPLAN software	2023	Slovakia	Retrospective	109 cochlea (56 pz)	7,3 (+-3,7)	/	Measured manually vs. measure with AUTO	/	To evaluate the new automatic measurement method	Cochlear diameter (A-value)<br>Height of the cochlea (H-value)<br>Width of the cochlear base (B-value)<br>CDL	Difference in CDL measurement and in measurement time	There wasn't a significant difference in measurement of Choclear parameters; Time neeed to perform the measurements was reduced from 7 min to 1min.
Dillon *et al.*[55]	Effect of Place-Based Versus Default mapping Procedures on Masked Speech Recognition: simulation of Cochlear Implant	2021	United States of America	Prospective	25 pz	18-25	/	Default filter frequencies vs. place-based filter frequencies	Unilateral	Compare performance with default maps vs. experimental place-based map, in partecipans with normal earing	AID<br>Estimate cochlear place frequency for each electrode	Sentence recognition	Better performance with the placed-based maps than for the default maps for both the CI-alone and EAS simulation.

	Alone and Electric-Acoustic Stimulatio.												Adding acoustic low-frequency information resulted in a similar benefit for both maps.
Mertens *et al.* [54]	The smaller the frequency-to-place mismatch the better the hearing outcomes ini cochlear implant recipiens	2022	Belgiu m	Retrospect ive	39 pz	17-81	Postlingual deafness	/	Unilatera l	To investigate the effect of frequency-to-place mismatch; investigatin g if there is any correlation between Aid and CDL, and which is the mean deviation of the default frequency map.	AID<br>CDL<br>Estimate cochlear place frequency for each electrode	Speech perception Correlation between frequency-to-place mismatch and speech perception<br>AID Correlation between AID and CDL<br>Mean frequency shift	Significant linear correlation between the frequency-to-place mismatch and speech perception in noise 6 month after CI. The smaller the frequency-to-place mismatch, the better the initial speech perception in noise. The significant effect disappeared after 12 months. AID from 458° to 642°. mean of CDL 32,96. Correlation: 0,47; mean

													frequancy shift: from 1,03 to 1,44.
Mertens *et al.*[34]	Prediction of the Cochlear Implant Electrode Insertion Depth: Clinical Applicability of two Analytical Cochlear Models (2020)	2020	Belgiu m	Retrospect ive	46 pz	mean 56 (6-81)	/	pre-operative AID vs post-operative AID	unilateral	To compare the clinical applicability of the Escudè and ECA formula using OTOPLAN to predict the AID; to compare the AID calculated with OTOPLAN to the prediction based upon a two-dimensional CT image.	Cochlear diameter (A-value)<br>Width of the cochlear base (B-value)<br>CDL<br>AID	Intra- and Inter-observer agreement<br>Validation of AID prediction	The use of a new planning software taht allows three-dimensional handling, integrating the diameter and width of the basal turn (ECA formula), resulted in the most accurate predictions of the AID.
Canfarott a *et al.*[50]	Incidence of Complete Insertion in Cochlear Implant Recipients of Long Lateral Wall Arrays	2020	United States of Americ a	Retrospect ive	51 pz	23-87	/	complete insertion vs partial inserion	Unilatera l	To investigate the incidence of complete insertions among patients implanted with 31,5 mm flexible arrays and whether complete insertion is	Cochlear diameter (A-value)<br>Width of the cochlear base (B-value)<br>CDL	Partial insertion rate<br>AID<br>CDL	Complete insertion of a 31.5 mm flexible array is feasible in most cases and does not appear to be limited by the range of CDL.

										limited by cochlear duct lenght.			
Müller-Graff *et al.*[29]	Implementation of the secondary reconstructions of flat-panel volume cmputed tomography (fpVCT) and otological planning software for anatomically based cochlear implantation	2022	Germany	Retrospective	30 pz	57-64	/	MSCT in non-implanted ear vs. MSCT in implanted ears	Unilateral	To investigate the combination of fpVCT and otological planning software to improve the implementation of anatomically based cochlear implantation.	Cochlear diameter (A-value)<br>Width of the cochlear base (B-value)<br>CDL	Comparison of CDL measurements	The combination of $fpVCT_{(seco)}$ and OTOPLAN permits a simplified and more reliable analysis of the cochlea in the pre and postoperative setting. The combination of both systems will enable further progress in the development of an anatomically based CI.
Canfarotta et al.[46]	Influence of Age at Cochlear Implantation and Frequency-to-place Mismatch on Early Speech	2019	United States of America	Retrospective	48 pz	67,4 (42-95)	Postligual deafness	/	Unilateral	To investigate the influence of mismatch and age at implantation on speech recognition within the initial 6	AID<br>Spiral ganglion place frequency	AID<br>Frequancy-to-place mismatch<br>Postoperative Speech Recognition	Younger adult CI recipients experienced more rapid growth in speech recognition during the initial 6 months

	Recognition in Adults									months of CI use.			post-activation. Greater degrees of frequency-to-place mismatch were assiciated with poorer performance, yet older listeners were not particularly susceptible to this effect.
Breitsprecher et al.[15]	CT imaging-based approaches to cochlear duct length estimation - a human temporal bone study	2021	Germany	Prospective	20 pz	/	/	CDL using 3D reconstruction with a 3D software CDL using the A-value method CDL using OTOPLAN	Unilateral	To detrmine the most reliable radiological imaging method and imaging processing software for measuring CDL from clinical routine imagnig and to predict the AID.	Cochlear diameter (A-value) Height of the cochlea (H-value) Width of the cochlear base (B-value) CDL	CDL	Alla approaches would have led to an electrode choice of rather too short electrodes. Concerning treatment decisions based on CDL measurements, the OTOPLAN-based method has to be recommended.

Almuhawas *et al.*[35]	Age as a factor of growth in mastoid thickness and skull width	2020	Saudi Arabia	Retrospective	92	<20years and >20 years (range 6months -79 years)	Normal; Inner ear malformation: hypoplasia, incomplete partition type I, II and III, enlarged vestibular aqueduct syndrome, ossified cochlea	normal cohclea vs malformed	/	To understand the growth rate of mastoid thickness and skull width associated with the age for both normal and malformed inner-ear anatomy groups. Also, to determine if there is any mathematical relation between cochlear size as measured by the ‘‘A’’ value against the age, mastoid thickness, and skull width.	Mastoid thickness Cochlear diameter (A-value) Skull width	Mastoid thickness Skull width	Mastoid thickness and skull width increased with age, while the cochlear size was independent of age, mastoid thickness, and the size of the skull
Andersen et al.[37]	Segmentation of Temporal Bone Anatomy for Patient-Specific Virtual Reality Simulation	2020	United States of America	Retrospective	9pz	3 months - 12 years	/	OTOPLAN vs. manual segmentation	/	To explore different approaches to segmentation of temporal bone surgical anatomy fo	Cochlear diameter (A-value) Height of the cochlea (H-value) Width of the cochlear base (B-value)	Segmented volumes Segmentations time	The automated segmentation algorithm currently offers the most flexible and feasible approach.

										patient-specific VR simulation.			
Dhanasingh *et al.*[30]	A novel three-step process for the identification of inner ear malformation types	2022	Saudi Arabia	Retrospective	112 ears	/	Inner ear malformation	/	/	Visualizing inner-ear systematically in both cochlear view (oblique coronal plane) and in mid-modiolar section (axial plane) and following three sequential steps simplifies, identification of inner-ear malformation types.	Cochlear diameter (A-value)<br>Width of the cochlear base (B-value)<br>Angular turn of the LW<br>Mid-modiolar section	Cochlear diameter (A-value)<br>Width of the cochlear base (B-value)<br>Angular turn of the LW<br>Mid-modiolar section	The systematic application of the three-step process proposed in this study is a novel method in the identification of IEM types. The visualizing inner ear in both cochlear view and the mid-modiolar section enables to capture every key anatomical structure of the inner ear in the identification of anatomical types.

Canfarotta et al.[48]	Insertion Depth and Cochlear Implant Speech Recognition Outcomes: A Comparative Study of 28- and 31.5-mm Lateral Wall Arrays	2022	United States of America	Retrospective	75 pz	65,0 (28mm) - 63,6 (31,5mm)	Unknown Meniere's Noise induced Usher's syndrome Temporal bone fracture	Array lenght: 28mm vs. 31.5mm	Unilateral	To compare speech recognition outcomes between cochlear implant (CI) recipients of 28- and 31.5-mm lateral wall electrode arrays, and to characterize the relationship between angular insertion depth (AID) and speech recognition.	AID	Consonant-nucleus-consonant (CNC) word recognition	Cochlear implant recipients implanted with a 31.5-mm array experienced better speech recognition than those with a 28-mm array at 12 months post-activation. Deeper insertion of a lateral wall array appears to confer speech recognition benefit up to ~600°, with a plateau in performance observed thereafter
Li et al. [31]	Analysis of Cochlear Parameters in Paediatric Inner Ears with Enlarged Vestibular Aqueduct and Patent Cochlea	2022	China	Retrospective	247 ears	0.0-12.8		patent cochlea VS enlarged vestibular aqueduct VS enlarged vestibular aqueduct with incomplete		(a) assess the cochlear dimensions in Chinese paediatric CI candidates with fully developed patent cochleae and with	The A-value (diameter), B-value (width), and H-value (height) were measured using OTOPLAN (version 1.2).	different cochlear anatomies using CT images and 3D images in both axial and coronal view. Data were analysed as per side of	A significant difference was found for the A value and B value between the patent cochleae and EVA-only and between

								partition type II		EVA using the OTOPLAN software (b) analyse the differences between side of the ear, sex, and type of malformation.		the ear (left/right), sex, and type of malformation	the patent cochleae and EVA with IP II. . The basal turn of the cochlea may be smaller in EVA cases than in the patent cochleae. Electrode selection should be adjusted accordingly
Zhu et al. [45]	The effect of cochlear size on electrically evoked auditory brainstem responses in deaf children	2023	China	Retrospective	75 pz	0.67-18	severe or profound bilateral sensorineural hearing loss (SNHL)	no inner ear malformations (IEMs) VS Mondini malformation	first CI	to investigate the relationship between cochlear size and auditory conduction function in deaf children with no IEMs and those with Mondini malformation.	Basal cochlear diameter, cochlear width, cochlear height and CDL,	Correlations between EABRs and cochlear sizes were analyzed.	The EABR thresholds and/or latencies were negatively correlated with the basal cochlear diameter, cochlear width and/or cochlear duct length in both patients without IEMs and those with Mondini malformation. A larger cochlear

													size appears to be associated with better auditory conduction function.
